# Association between preterm brain injury and exposure to chorioamnionitis during fetal life

**DOI:** 10.1038/srep37932

**Published:** 2016-12-01

**Authors:** Devasuda Anblagan, Rozalia Pataky, Margaret J. Evans, Emma J. Telford, Ahmed Serag, Sarah Sparrow, Chinthika Piyasena, Scott I. Semple, Alastair Graham Wilkinson, Mark E. Bastin, James P. Boardman

**Affiliations:** 1 MRC Centre for Reproductive Health, University of Edinburgh, Queen’s Medical Research Institute, 47 Little France Crescent, Edinburgh EH16 4TJ, UK; 2 Centre for Clinical Brain Sciences, University of Edinburgh, Chancellor’s Building, 49 Little France Crescent, Edinburgh EH16 4SB, UK; 3 Department of Pathology, Royal Infirmary of Edinburgh, 51 Little France Crescent, Edinburgh, EH16 4SA, UK; 4 Centre for Cardiovascular Science, University of Edinburgh, Queen’s Medical Research Institute, 47 Little France Crescent, Edinburgh EH16 4TJ, UK; 5 Clinical Research Imaging Centre, University of Edinburgh, 47 Little France Crescent, Edinburgh EH16 4TJ, UK; 6 Department of Radiology, Royal Hospital for Sick Children, 9 Sciennes Road, Edinburgh, EH9 1LF, UK

## Abstract

Preterm infants are susceptible to inflammation-induced white matter injury but the exposures that lead to this are uncertain. Histologic chorioamnionitis (HCA) reflects intrauterine inflammation, can trigger a fetal inflammatory response, and is closely associated with premature birth. In a cohort of 90 preterm infants with detailed placental histology and neonatal brain magnetic resonance imaging (MRI) data at term equivalent age, we used Tract-based Spatial Statistics (TBSS) to perform voxel-wise statistical comparison of fractional anisotropy (FA) data and computational morphometry analysis to compute the volumes of whole brain, tissue compartments and cerebrospinal fluid, to test the hypothesis that HCA is an independent antenatal risk factor for preterm brain injury. Twenty-six (29%) infants had HCA and this was associated with decreased FA in the genu, cingulum cingulate gyri, centrum semiovale, inferior longitudinal fasciculi, limbs of the internal capsule, external capsule and cerebellum (*p* < 0.05, corrected), independent of degree of prematurity, bronchopulmonary dysplasia and postnatal sepsis. This suggests that diffuse white matter injury begins *in utero* for a significant proportion of preterm infants, which focuses attention on the development of methods for detecting fetuses and placentas at risk as a means of reducing preterm brain injury.

Globally, preterm birth affects around 10% of deliveries and is a leading cause of neurodevelopmental impairment^1^. Adverse outcome is strongly associated with a phenotype that combines diffuse white matter injury and reduced connectivity of developing neural systems apparent on neonatal brain magnetic resonance imaging (MRI), with cognitive impairment and educational under-attainment in childhood^2^,^3^,^4^,^5^. This phenotype is partly explained by co-morbidities such as bronchopulmonary dysplasia (BPD)^6^ and postnatal sepsis^7^, and is influenced by nutritional^8^ and genetic factors^9^. Pre-clinical and epidemiological studies demonstrate a clear association between abnormal systemic inflammation at a critical point in development and preterm brain injury (for review see^10^).

Chorioamnionitis (infection/inflammation of the amniotic fluid, membranes, placenta and/or decidua) affects around 40–80% of very preterm deliveries and it can initiate a fetal inflammatory response that is injurious to the developing brain and other organs^11^,^12^. In meta-analyses, chorioamnionitis is associated with cystic periventricular leukomalacia and cerebral palsy in preterm infants^13^, but there is uncertainty about its contribution to the more common preterm phenotype (diffuse white matter injury), and its importance in relation to co-morbidities. Some of these uncertainties may be attributable to study designs that have used variable case definitions (clinical or histopathological diagnostic criteria), and many studies were conducted before the era of quantitative neonatal brain MRI for defining cerebral outcome.

Diffusion MRI (dMRI) provides objective measures of white matter microstructure in the newborn that are altered in association with preterm birth: a consistent finding is that fractional anisotropy (FA), a biomarker linked to white matter microstructure, is reduced in white matter tracts of preterm infants at term equivalent age compared with term-born matched controls^14^,^15^. Tract-based Spatial Statistics (TBSS) is a powerful unbiased method for group-wise analysis of FA images derived from dMRI data^16^. It has been applied to neonatal dMRI to map microstructural change in white matter tracts of preterm infants at term equivalent age^17^, to identify clinical risk factors for altered brain development^6^, to detect tissue effects of neuroprotective treatment strategies^18^, and it may have a role in early risk stratification because it predicts neurodevelopmental outcome^2^,^5^. Computational analysis of structural MRI data enables the calculation of brain volume (whole brain or tissue compartments) and is useful for identifying factors associated with growth deficits^8^,^19^, and for longitudinal modeling of growth in early life^20^,^21^.

We combined histologic classification of chorioamnionitis with neonatal brain MRI, and used TBSS and computational morphometry to test the hypothesis that HCA is associated with altered white matter microstructure and brain volume in preterm infants at term equivalent age.

## Results

### Patients

Placental histopathology and brain dMRI were acquired from 90 infants born preterm: 26 (29%) had histologic chorioamnionitis and 64 (71%) had no evidence of chorioamnionitis. Table 1 summarizes the demographic and clinical details of the two groups.

Preterm infants with HCA had a lower GA at birth (*p* = 0.001), and the prevalence of prolonged rupture of membranes (>24 hours before delivery) was higher in this group (69% versus 8%). The proportion of infants exposed to antenatal MgSO_4_ for the purpose of fetal neuroprotection was greater in the HCA group: 69% versus 44% (*p* = 0.037). There was no significant difference in antenatal steroid exposure between the groups. Five out of 90 participants were treated for necrotizing enterocolitis, and three required treatment for retinopathy of prematurity. Postnatal somatic growth, measured as the difference between birth weight z-score and weight at scan z-scores, did not differ significantly between the two groups: the mean difference (SD) in weight z-score for the HCA group was −0.98 (1.07) versus −0.67 (1.06) for preterm infants without HCA exposure (t = 1.247, *p* = 0.216).

### Placental histopathology

26 of 90 (29%) placental plates showed patterns of inflammatory reaction on the maternal side, and 20 of these showed an inflammatory response of the cord and/or the umbilical vessels. The patterns of maternal/fetal inflammation are shown in the Table 2.

### Magnetic resonance imaging

49 infants (54%) had abnormal white matter using the classification system described by Woodward *et al*.^22^, and 8 infants (9%) had punctate white matter lesions. One infant had a solitary cyst in left peritrigonal white matter (diameter 5 mm).

### White matter correlates of histologic chorioamnionitis

**Figure 1:**
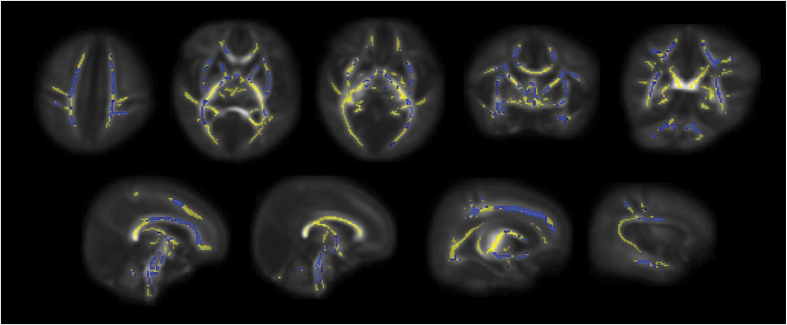
Mean FA skeleton (yellow) overlaid on the mean FA map in axial, coronal and sagittal planes. Voxels demonstrating significantly lower FA in preterm infants at term equivalent who had been exposed to HCA are overlaid in blue.

**Figure 2:**
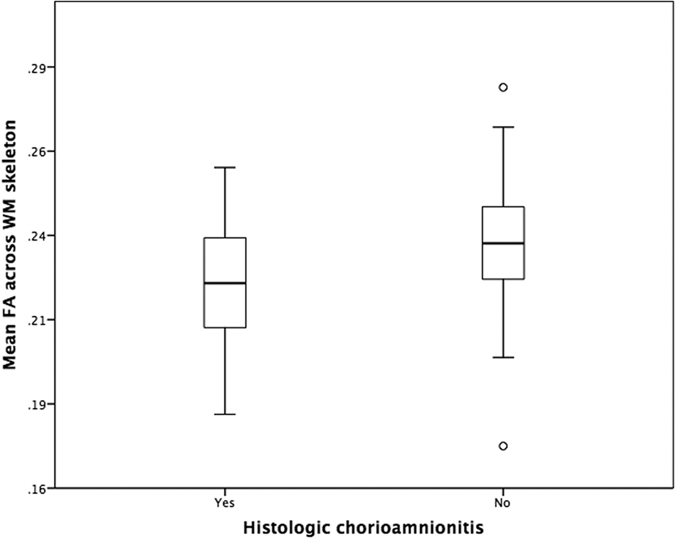
Mean FA across the white matter skeleton of preterm infants grouped by presence of histologic chorioamnionitis.

The findings are provided corrected for GA at birth, GA at image acquisition, BPD and postnatal sepsis. Preterm infants exposed to HCA had decreased FA in the genu of corpus callosum, cingulum cingulate gyri, centrum semiovale, corticospinal tracts (CST), inferior longitudinal fasciculi (ILF), left arcuate, anterior and posterior limbs of internal capsule, external capsule and cerebellum (Fig. 1); *p* < 0.05 corrected. Skeleton-wide FA was 5.3% lower in preterm infants with HCA compared to infants born without this exposure; *p* = 0.006 (FA in the range of 0.182–0.255 for infants with HCA and 0.172–0.279 for infants without HCA exposure, respectively), (Fig. 2).

### Histologic chorioamnionitis and brain tissue volume

**Figure 3:**
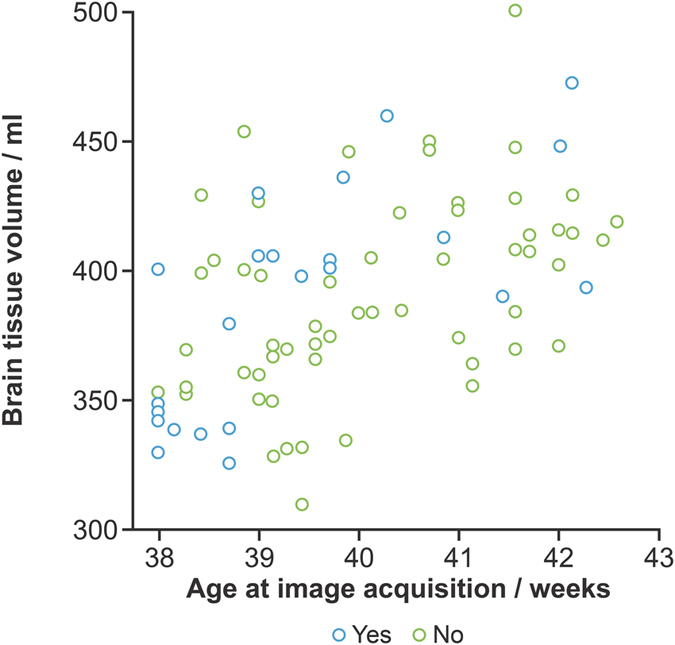
Brain tissue volume at term equivalent age for each participant colour coded by presence of HCA (blue) or absence of HCA (green).

Structural MRI data were available for 81 of the 90 participants; nine structural MRI data were not useable due to movement artefact. Whole brain tissue volume increased over the range of gestational age at image acquisition (Fig. 3), but there was no difference in brain tissue volume attributable to HCA: mean 388.9 ml (SD 44.0) for 23 infants with HCA versus mean 391.4 ml (SD 37.4) for 58 infants without the exposure. There were no significant differences in the volume of any tissue compartment or cerebrospinal fluid between the groups (Table 3).

## Discussion

By combining histological diagnosis of chorioamnionitis with quantitative brain MRI, we have shown that inflammation *in utero* contributes to altered microstructure in major white matter tracts of preterm infants at term equivalent age. The effect was independent of age at image acquisition and known predictors of preterm brain injury and poor neurodevelopmental outcome including degree of prematurity, BPD and postnatal sepsis.

Meta-analysis has shown that HCA is a significant predictor of cerebral palsy (CP)^13^, and HCA has been associated with intraventricular haemorrhage^23^, brain imaging abnormalities^24^, and cystic periventricular leukomalacia^25^. However, the role of confounding by gestational age at birth and co-morbid conditions on these associations is unclear; and in some studies chorioamnionitis has no adverse effect on neurodevelopmental outcome^26^,^27^. Such differences may be explained by the heterogeneity of HCA, which encompasses a range of grades and stages of maternal and fetal inflammation that have variable effects on the fetal inflammatory response. A strength of this study is that 77% of infants classified as HCA-exposed had fetal vasculitis affecting the cord (funisitis) or chorionic plate, which is the histological indicator of fetal inflammatory response syndrome (FIRS) since it reflects transmigration of fetally derived neutrophils to bacteria in the amniotic fluid^28^.

The fetal inflammatory response is characterized by increased concentration of pro-inflammatory cytokines in plasma^29^. During postnatal life these mediate brain injury by several mechanisms including: increased permeability of the blood brain barrier to cytotoxic proteins^30^; activation of microglia^31^; dysmaturation of the oligodendrocyte lineage and ultimately hypomyelination^32^; neuronal injury and cell death^33^; modification of the endogenous stem cell populations^10^; generation of reactive oxygen and nitrogen species^32^; sensitization of the brain to subsequent hypoxic-ischaemic insults^34^; and activation of the coagulation cascade^35^. After delivery, systemic inflammation due to postnatal bloodstream infection or necrotizing enterocolitis is a powerful predictor of neurodisability among preterm infants^7^, and MRI studies suggest that the neural substrate includes injury to the white matter^36^,^37^. Our data suggest that *antenatal* exposure to systemic inflammation contributes to the prevailing form of preterm brain injury. This is consistent with a previous observation that elevated cytokines and CD45RO^+^ T lymphocytes in umbilical cord blood are associated with overt cerebral lesions on MRI very soon after birth^38^. MRI acquisition in the early post-partum period after very preterm birth (around 30 weeks gestational age) is feasible and recent advances in post-processing techniques enable the evaluation of diffuse white matter injury, connectivity measures, cortical maturation and growth trajectories at this early stage^39^,^40^,^41^,^42^,^43^,^44^. These approaches could be combined with collateral biological information about fetal/maternal inflammation to investigate specific antenatal determinants of abnormal brain development.

We used TBSS to survey the white matter skeleton for group-wise differences that might be associated with HCA because of its sensitivity for detecting alterations in FA in the newborn period, and because of its predictive value for later outcome^2^,^5^. FA is considered a robust marker of tract microstructure that reflects fibre density, axonal diameter, wrapping by pre-myelinating oligodendrocytes and myelination. These data suggest that infants exposed to HCA have less coherently organised and more immature fibre tracts at term equivalent age compared with preterm infants without HCA-exposure. We found no difference in whole brain tissue volume, or the volumes of tissue class compartments between the groups, which is consistent with previous observations that preterm brain injury is characterized by reduced connectivity of neural systems rather than a global failure of brain growth for the majority of preterm infants^19^,^45^. BPD and intrauterine growth restriction are associated with reduced brain volumes, but the prevalence of these was similar between groups^8^,^19^. In previous work we showed that preterm infants with elevated ADC values in white matter had focal tissue volume reduction in deep grey matter nuclei compared with healthy term controls born at term^46^,^47^, but there was no difference in deep grey matter structure when the preterm group was compared with preterm infants without white matter injury. This is consistent with the findings of the current study, and may be explained by smaller differences in water diffusion parameters between preterm subgroups, compared with the differences reported between preterm infants and healthy controls^17^.

Our study has some limitations. Firstly, although 77% of the HCA group had fetal vasculitis, which is indicative of FIRS, we did not analyze cord blood so further study is required to uncover the specific immune mediators that might link HCA with white matter injury, and also to investigate susceptibility in infants without FIRS. Secondly, the study was not powered to investigate the role of multiple placental lesions, which could act synergistically to increase fetal risk. Thirdly, the diagnosis of neonatal sepsis is imprecise; we overcame this by using a rigorous clinical protocol that meets international bench-marking standards (Vermont Oxford Network) but it is possible that some cases were misclassified. In future, integrating quantitative imaging data with information about the host response to infection/inflammation provided by pathway analysis may offer new insights into the timing and drivers of inflammation-induced perinatal brain injury.

In conclusion, these results suggest that the pathway to white matter injury originates *in utero* for a significant proportion of preterm infants exposed to HCA during fetal life. Future research could focus on the development of antenatal tests to identify HCA and fetuses at risk of neuroinflammation; and placental histopathology may have a role in risk stratification for trials of immune-modulatory therapies designed to improve outcome.

## Methods

### Participants

The study was conducted according to the principles of the Declaration of Helsinki, and ethical approval was obtained from the UK National Research Ethics Service (South East Scotland Research Ethics Committee). Written parental informed consent was obtained for all subjects. The cohort consisted of preterm infants born at <33 completed weeks’ gestational age (GA) who received care at the Royal Infirmary of Edinburgh between July 2012 and May 2015, who underwent brain MRI at term equivalent age and whose placentas were available for histologic examination. Exclusion criteria were infants with congenital infection, chromosomal abnormalities, post-haemorrhagic ventricular dilatation, porencephalic cysts and cystic periventricular leucomalacia.

Postnatal sepsis was defined by: 1) positive blood culture growing pathogenic bacteria; *or* 2) blood cultures negative or positive for coagulase negative staphylococcus (CoNS) *plus* generalized signs of infection *plus* physician decision to treat with antibiotics for 5 days or more. BPD was defined by the requirement for supplemental oxygen at 36 weeks’ GA.

Postnatal somatic growth was described in terms of change in weight z-score between birth and scan, calculated using Intergrowth-21^st^ reference standards for preterm infants^48^.

### Placental histopathology

**Figure 4:**
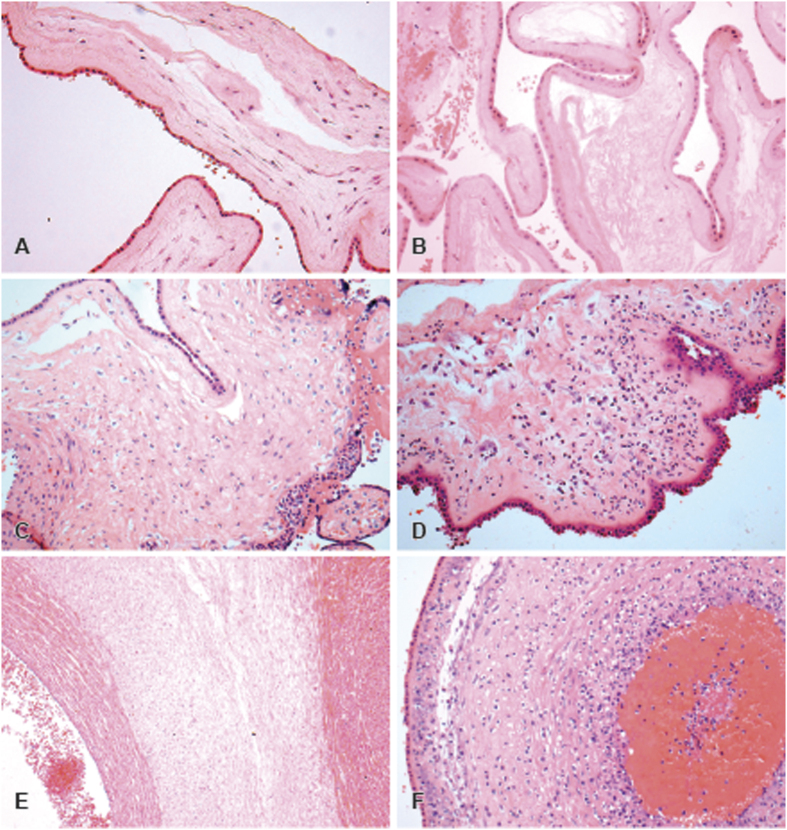
Histologic features of normal membranes and reaction patterns related to maternal inflammatory response to amniotic fluid infection. (**A,B)** Normal appearances of the placental membranes with no signs of inflammation. (**C)** Acute chorionitis (Stage 1) patchy-diffuse accumulations to neutrophils in the subchorionic plate fibrin. (**D)** Acute chorioamnionitis (Stage 2). Histologic features of non-inflamed cord and reaction patterns of fetal inflammatory response to amnionitic fluid infection: (**E**) Normal appearances of umbilical vein and artery and Wharton tissue. (**F**) Diffuse funisitis along with umbilical vasculitis.

After delivery, placentae were formalin fixed and stored at 4 °C before sampling. The placentae were sampled according to a standardized protocol; distal and proximal sections of cord (the proximal section being taken at 1.5 cm from above the fetal surface), a roll of extraplacental membranes starting at the point of rupture and 4 full thickness sections from each quadrant. All were stained with Haematoxylin and Eosin. Slides were examined by a single experienced placenta pathologist (ME) with no knowledge of the MRI findings. Placental reaction patterns were reported according to site and degree of inflammation, using the structure proposed by Redline and colleagues^49^. The chorioamnionitis group included cases which demonstrated an inflammatory response in the placental membranes of any grade or stage, whilst the non-chorioamnionitis group demonstrated no inflammatory response. Features of normal and inflamed placental tissue are illustrated in Fig. 4.

### MRI acquisition

MRI was performed on a Siemens Magnetom Verio 3 T system (Siemens, Healthcare Gmbh, Erlangen, Germany) using a 12-channel matrix phased array head coil. All infants were scanned axially to acquire: 3D T1-weighted MPRAGE volume (~1 mm^3^ resolution), T2-weighted STIR (~0.9 mm^3^ resolution), T2-weighted FLAIR (~1 mm^3^ resolution), and dMRI (11 T2- and 64 diffusion encoding direction (b = 750 s/mm^2^) single-shot spin-echo echo planar imaging (EPI) volumes with 2 mm isotropic voxels data, TE = 106 ms and TR = 7300 ms) data. Images were reported by a radiologist with experience in neonatal MRI (AGW).

Infants were scanned in natural sleep with monitoring of pulse oximetry, electrocardiography and temperature. A neonatal doctor and a research nurse were present during image acquisition. For ear protection, flexible earplugs and neonatal earmuffs (MiniMuffs, Natus Medical Inc., CA) were used.

Structural MRI scans were scored using the system described by Woodward *et al*.^22^. In summary, a white matter injury (WMI) score was calculated by adding sub-scores across 5 domains, each evaluated by a 3 point grading scale: white matter signal abnormality, periventricular white matter volume loss, presence of cystic abnormalities, ventricular dilatation, and thinning of corpus callosum. A grey matter injury (GMI) score was calculated by adding sub-scores from 3 domains: cortical abnormalities, quality of gyral maturation, and size of subarachnoid space. Therefore WMI scores ranged between 5 and 15, and GMI scores between 3 and 9. WMI scores were classified as per the original system from Woodward *et al*.^22^, i.e. ≤6 is normal and >6 is abnormal. GMI scores were classified as abnormal if ≤4, and normal as >4, using the modification described by Leuchter *et al*.^50^. The presence of punctate white matter lesions (high signal intensity on T1-weighted imaging and low signal on T2-weighted) was also recorded.

### Image data analysis

#### Tract-based Spatial Statistics

dMRI data were preprocessed using FSL tools (FMRIB, Oxford, UK; http://www.ndcn.ox.ac.uk/divisions/fmrib). This included brain extraction, and removal of bulk infant motion and eddy current induced artifacts by registering the diffusion-weighted volumes to the first T2-weighted EPI volume for each subject. Using DTIFIT, FA maps were generated for every subject.

TBSS analysis was performed using a pipeline that was optimized for neonatal dMRI data^6^. An average FA map and mean FA skeleton (thresholded at FA > 0.15) were created from the aligned data, representing the center of all white matter tracts common to both groups. Statistical comparison between groups born with and without exposure to chorioamnionitis was performed with FSL’s Randomise using a general linear univariate model, with GA at birth, GA at image acquisition, BPD and postnatal sepsis listed as covariates. All FA data were subject to family-wise error correction for multiple comparisons following threshold-free cluster enhancement (TFCE) and are shown at *p* < 0.05^51^.

#### Volumetric analysis

The brain tissue was separated from non-brain tissue using an atlas-based approach^21^. Images were corrected for field inhomogeneity using the N4 method (http://stnava.github.io/ANTs/). All images from the dataset were non-linearly aligned to the 40 weeks’ GA template from a neonatal brain 4D atlas^52^, which is the closest age-matched template to the mean age of the subjects in this study. Then, an Expectation–Maximization framework was used to segment the brain into: brainstem, cerebellum, cortex, cerebrospinal fluid, deep gray matter and white matter; where probabilistic spatial priors of each tissue were provided by the 4D atlas. Volumes were calculated for each individual tissue, and total brain tissue volume was calculated by subtracting cerebrospinal fluid from the whole brain segmentation.

### Statistics

Student’s t-test or the Mann-Whitney test was used to investigate differences in clinical and demographic variables between infants with (n = 26) and without chorioamnionitis (n = 64), and chi-square test or Fisher’s exact test was used to compare proportions. Statistical analysis was performed using SPSS v21.0 (SPSS Inc, Chicago, IL).

## Additional Information

**How to cite this article**: Anblagan, D. *et al*. Association between preterm brain injury and exposure to chorioamnionitis during fetal life. *Sci. Rep.* **6**, 37932; doi: 10.1038/srep37932 (2016).

**Publisher's note:** Springer Nature remains neutral with regard to jurisdictional claims in published maps and institutional affiliations.

## Figures and Tables

**Table 1:** Demographic and clinical features of preterm infants with and without chorioamnionitis.

		Histologic Chorioamnionitis (n = 26)	No Histologic Chorioamnionitis (n = 64)	*p* value
Mean GA age at birth/weeks (range)		27^+6^ (23^+2^–30^+4^)	29^+4^ (25^+0^–32^+6^)	0.001
Mean birth weight/g (SD)		1088 (550–1525)	1180 (670–1635)	0.106
Mean birth weight z-score (SD)		0.17 (0.49)	−0.39 (0.94)	0.007
Mean GA at scan/weeks (range)		39^+4^ (38^+0^–42^+2^)	40^+2^ (38^+0^–42^+5^)	0.012
Mean weight z-score at scan (SD)		−0.71 (1.16)	−1.07 (1.08)	0.177
Postnatal sepsis, n (%)	Any	16 (62)	22 (36)	0.033
	Early onset*	8 (31)	7 (11)	0.031
	Late onset**	10 (38)	20 (31)	0.623
Necrotizing enterocolitis, n (%)		2 (8)	3 (5)	0.624
Gender (M:F)		12:14	34:30	0.569
BPD, n (%)		8 (31)	17 (27)	0.436
Antenatal MgSO_4_, n (%)		18 (69)	28 (44)	0.037
Antenatal corticosteroid, n (%)		23 (88)	45 (70)	0.104

*Postnatal sepsis <72 hours after birth; **postnatal sepsis >72 hours after birth.

**Table 2:** Patterns of inflammatory response in 26 cases with histologic chorioamnionitis.

Fetal inflammatory responses					Maternal inflammatory responses					
Vasculitis (Stage 1)		Vasculitis 1 or more vessels (Stage 2)		Funisitis (Stage 3)	Chorionitis (Stage1)		Chorioamnionitis (Stage2)		Necrotising chorioamnionitis (Stage 3)	Breeches the chorion -Intervillositis
1 = Yes, 0 = No	Grade	1 = Yes, 0 = No	Grade		1 = Yes, 0 = No	Grade	1 = Yes, 0 = No	Grade	1 = Yes, 0 = No	1 = Yes, 0 = No
1	1	0		0	0		1	1	0	1
0		1	2	1	0		1	2	0	1
0		1	2	1	1	1	1	2	1	1
0		0		0	1	1	0		0	0
0		1	2	1	0		1	2	1	1
1	1	0		0	1	1	0		0	1
1	2	0		1	1	2	0		0	1
0		0		0	1	1	0		0	0
0		1	1	1	0		1	2	1	1
0		1	1	1	1	1	0		0	1
0		1	2	1	0		1	2	1	0
0		1	1	1	1	2	0		0	1
0		1	2	1	1	1	0		0	1
0		1	2	1	0		1	2	1	0
0		0		0	1	1	0		0	0
0		1	1	1	0		1	1	1	1
0		1	2	1	0		1	1	0	1
0		1	1	1	0		1	2	0	1
0		1	2	1	1	2	0		1	1
0		1	1	1	0		1	2	0	0
0		0		0	1	1	0		0	0
0		0		0	1	1	0		0	1
0		0		0	1	1	0		0	0
0		1	1	1	0		1	2	0	1
0		1	1	1	0		1	1	0	1
1	1	0	0	1	0		0		0	0

**Table 3:** Mean (SD) brain tissue and cerebrospinal fluid volumes at term equivalent age.

	Mean volume (SD) / ml (histologic chorioamnionitis)	Mean volume (SD) / ml (no histologic chorioamnionitis)	*p*-value
White matter	138.8 (14.2)	138.4 (14.6)	0.70
Deep grey matter	5.2 (0.86)	5.3 (0.80)	0.19
Cortical grey matter	169.2 (24.6)	173.4 (19.7)	0.42
Cerebellum	22.7 (3.8)	24.5 (3.3)	0.06
Brainstem	5.2 (0.9)	5.2 (0.8)	0.70
Cerebrospinal fluid	79.8 (17.1)	75.6 (18.0)	0.32
